# Expression of human mutant cyclin dependent kinase 4, Cyclin D and telomerase extends the life span but does not immortalize fibroblasts derived from loggerhead sea turtle (*Caretta caretta*)

**DOI:** 10.1038/s41598-018-27271-x

**Published:** 2018-06-20

**Authors:** Tomokazu Fukuda, Takahiro Eitsuka, Kenichiro Donai, Masanori Kurita, Tomomi Saito, Hitoshi Okamoto, Kodzue Kinoshita, Masafumi Katayama, Hiroshi Nitto, Takafumi Uchida, Manabu Onuma, Hideko Sone, Miho Inoue-Murayama, Tohru Kiyono

**Affiliations:** 1 Graduate School of Science and Engineering, Iwate University, Morioka, Japan; 2 Soft-Path Engineering Research Center (SPERC), Iwate University, Morioka, Japan; 3 Wildlife Genome Collaborative Research Group, National Institute for Environmental Studies, Tsukuba, Ibaraki, Japan; 4 Graduate School of Agricultural Science, Tohoku University, Sendai, Miyagi Japan; 5 Port of Nagoya Public Aquarium, Nagoya, Japan; 6 Usa Marine Biological Institute, Kochi University, Tosa, Kochi, Japan; 7 Wildlife Research Center, Kyoto University, Kyoto, Japan; 8 Ecological Genetics Analysis Section, Center for Environmental Biology and Ecosystem, National Institute for Environmental Studies, Tsukuba, Ibaraki, Japan; 9 Environmental Exposure Research Section, Center for Environmental Risk Research, National Institute for Environmental Studies, Tsukuba, Ibaraki, Japan; 10 Division of Carcinogenesis and Cancer Prevention, National Cancer Center Research Institute, Tsukiji, Chuo-ku, Tokyo, 104-0045 Japan

## Abstract

Conservation of the genetic resources of endangered animals is crucial for future generations. The loggerhead sea turtle (*Caretta caretta***)** is a critically endangered species, because of human hunting, hybridisation with other sea turtle species, and infectious diseases. In the present study, we established primary fibroblast cell lines from the loggerhead sea turtle, and showed its species specific chromosome number is 2*n* = 56, which is identical to that of the hawksbill and olive ridley sea turtles. We first showed that intensive hybridization among multiple sea turtle species caused due to the identical chromosome number, which allows existence of stable hybridization among the multiple sea turtle species. Expressions of human-derived mutant Cyclin-dependent kinase 4 (CDK4) and Cyclin D dramatically extended the cell culture period, when it was compared with the cell culture period of wild type cells. The recombinant fibroblast cell lines maintained the normal chromosome condition and morphology, indicating that, at the G1/S phase, the machinery to control the cellular proliferation is evolutionally conserved among various vertebrates. To our knowledge, this study is the first to demonstrate the functional conservation to overcome the negative feedback system to limit the turn over of the cell cycle between mammalian and reptiles. Our cell culture method will enable the sharing of cells from critically endangered animals as research materials.

## Introduction

The loggerhead sea turtle is a critically endangered species, mainly because of human activity. Oil pollution of coastal areas, such as Deepwater Horizon oil well explosion in 2010, has caused serious damage to many sea turtle species, including the loggerhead^1^. Moreover, illegal hunting for turtle oil and meat continues. In addition to these human activities, a global outbreak of the fibropapilloma virus has been detected in sea turtles^2^, and is presenting a serious threat to the survival of these marine reptiles^3^.

In recent years, a high incidence of hybridisation has been reported among three species of sea turtle: the loggerhead, hawksbill and olive ridley sea turtle^4^. Lara-Ruiz *et al*. used mitochondrial DNA analysis to demonstrate that approximately 50% of hawksbill sea turtles at the Bahia coast of Brazil are hybrids with loggerheads^4^. Hybridisation between loggerhead and hawksbill sea turtles has also been detected in Japan^5^. Thus, there is a risk that the original sea turtle species may soon disappear. We need to clarify how this increased incidence of hybridisation has occurred among the multiple sea turtle species, since this information is important for the conservation of the original species of sea turtles and to maintain genetic diversity. Furthermore, Jensen *et al*. reported that dramatic biased sex ratio of sea turtle at Great Barrier Reef (GBR) in Australia. In brief, northern part of GBR, female ratio of sea turtle in Australia is around 99%, and only one % of males were detected^6^. Sex determination of sea turtle depend on the temperature sensitive, and does not have sex chromosome. The results of the Jensen *et al*. would be possibly explained by the results of global climate change, and would have significant impact for the reproductive efficiency and maintenance of sea turtle population. These situation indicates that sea turtle is one of the most critically endangered animals due to the human activity.

In 1976, the San Diego Zoo initiated the preservation of various types of biological specimens of endangered species, including germ cells, tissues and genomic DNA^7^. The “Frozen Zoo” initiated the starting of similar projects in other zoos in the United States and Europe. Conservation of endangered animals is crucial for future generations. However, the reproduction of wildlife is expensive and requires considerable human input. The purpose of the “Frozen Zoo” project is the conservation of biological resources for future generations. In the present study, we established normal fibroblast cell lines from loggerhead sea turtle tissue.

Furthermore, our research group previously reported that expression of human-derived mutant Cyclin-dependent kinase 4 (CDK4), Cyclin D and Telomerase Reverse Transcriptase (TERT) efficiently immortalises cells of various mammalian species, including human, bovine^8^, swine^9,10^, monkey^11^, prairie vole^12,13^ and midget buffalo^14^. We designated this recently developed method as K4DT, based on the identities of the introduced genes (mutant CDK4, Cyclin D and TERT). In the molecular evolution of animals, the amino acid sequences of the Cyclin proteins have been strongly conserved among the multiple species^15^. This background led us to propose the hypothesis that the expression of human mutant K4DT might also induce enhanced cell growth in reptiles, which would provide functional evidence that the basic mechanism controlling cellular proliferation is evolutionally conserved.

This study provides the first demonstration of the functional conservation from mammals to reptiles of cell cycle regulators. The application of cell cycle-related molecular genetics to wildlife science will enable the efficient establishment of cell lines from critically endangered animals, thereby providing research material for studies of genetic diversity and animal evolution.

## Results

### Optimisation of cell culture conditions for the primary cell lines from loggerhead sea turtle

**Figure 1:**
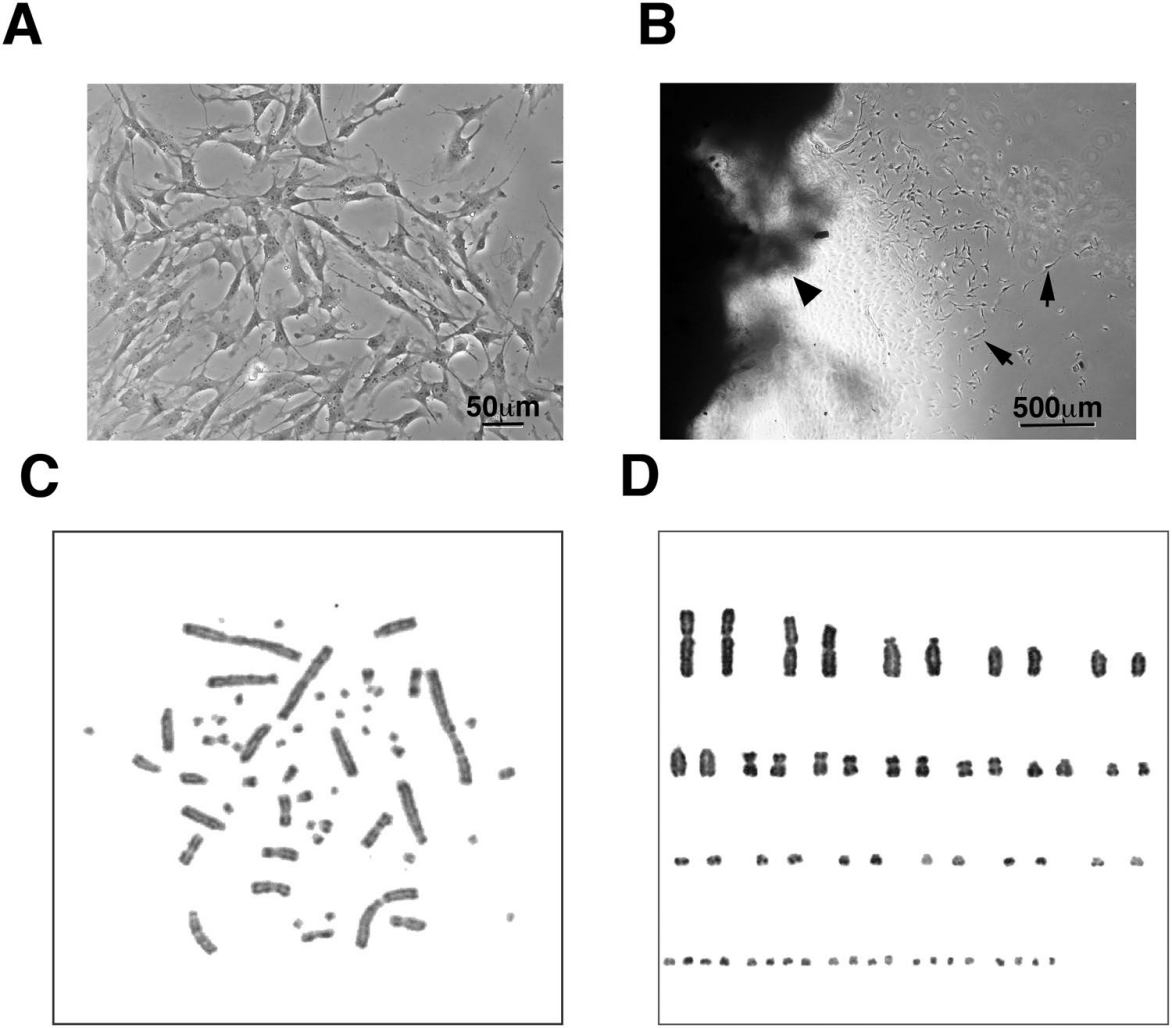
Primary cell culture of the loggerhead sea turtle under different conditions, and from frozen preserved tissue. (**A**) Cell morphology derived from the loggerhead sea turtle. Bar indicates 50 µm. (**B**) Primary cells obtained from the frozen preserved loggerhead tissue. Arrows indicate the primary cells. The arrowhead indicates the primary tissue. Bar indicates 500 µm. (**C**) Representative mitotic phase of the loggerhead cells. (**D**) Aligned chromosomes of the loggerhead cells. The result revealed a chromosome number of 2*n* = 56.

We previously used RPMI 1640 medium and a temperature of 26 °C for primary cell lines from the hawksbill sea turtle^16^. Our preliminary data indicated that hawksbill derived primary cells shows good growth in RPMI 1640, whereas primary cells of the loggerhead sea turtle does not (data not shown). Presently, we tested four different basal media—Dulbecco’s modified Eagle’s medium/Nutrient Mixture F-12 (DMEM/F12), RPMI 1640, Ham F12 and DMEM containing 10% fetal calf serum (FCS)—to determine the optimal conditions for the culture of primary cells of the loggerhead sea turtle. The primary cell number was highest in DMEM/F12, indicating that this medium provides the optimal conditions for primary cell culture of the loggerhead sea turtle. The obtained cells appeared spindle-shaped in the cell culture dish. This morphology indicated that the primary cells would be fibroblasts (Fig. 1A). Using the medium, we established 10 primary cell lines from 14 tissues, representing a 70% rate of success.

### Use of cryopreserved loggerhead sea turtle tissue to establish primary cell lines

For the successful establishment of primary cell lines from critically endangered animals, the cell culture materials must be transferred from the field to the laboratory under stable conditions, including transport of the primary samples. We hypothesised that transportation under cryopreserved conditions would be effective. To test this hypothesis, we acquired tissue samples by biopsy and immediately immersed small pieces of the tissues in the cryoprotective medium and froze the samples. The frozen primary tissues were stored for 6 months and were then thawed at 30 °C and used to start the primary culture. After 2 weeks, we observed the proliferation of primary cells (Fig. 1B). Thus, we concluded that cryopreserved loggerhead sea turtle tissue could be used as primary cell culture material.

### Karyotype analysis of the loggerhead sea turtle

We detected the chromosome number and banding pattern of the chromosome in the loggerhead sea turtle cell lines using G-banding. We analysed cells lines derived from two independent sea turtle individuals (Fig. 1C). Both turtles displayed a chromosome number of 2*n* = 56 (Fig. 1D). Our results differed from those of a previous study, which reported a chromosome number of 2*n* = 58^17^. To confirm the present results, we analysed >20 mitotic cells obtained from independent primary cultures of the two sea turtles. The chromosome number was consistently 2*n* = 56 (Figure S2C). Therefore, we concluded that the chromosome number of the loggerhead sea turtle is 2*n* = 56. We previously reported that the chromosome number of the hawksbill sea turtle was 2*n* = 56^18^. Based on our present and previous observations, we concluded that the loggerhead and hawksbill have an identical chromosome number.

### Gene transfer to loggerhead-derived cells

Primary cells undergo a limited number of cell divisions because of the Hayflick limit and cellular senescence^19^. However, it was recently described that the gene transfer of human-derived mutant CDK4, Cyclin D, and TERT into primary cells resulted in efficient cellular immortalisation, thereby enabling infinite cell division^10^. Furthermore, this immortalisation method was reported to maintain the normal chromosome condition and preserve the biological nature of the original cells^10^. Thus, we expected that this method might enable us to establish immortalised sea turtle cells.

**Figure 2:**
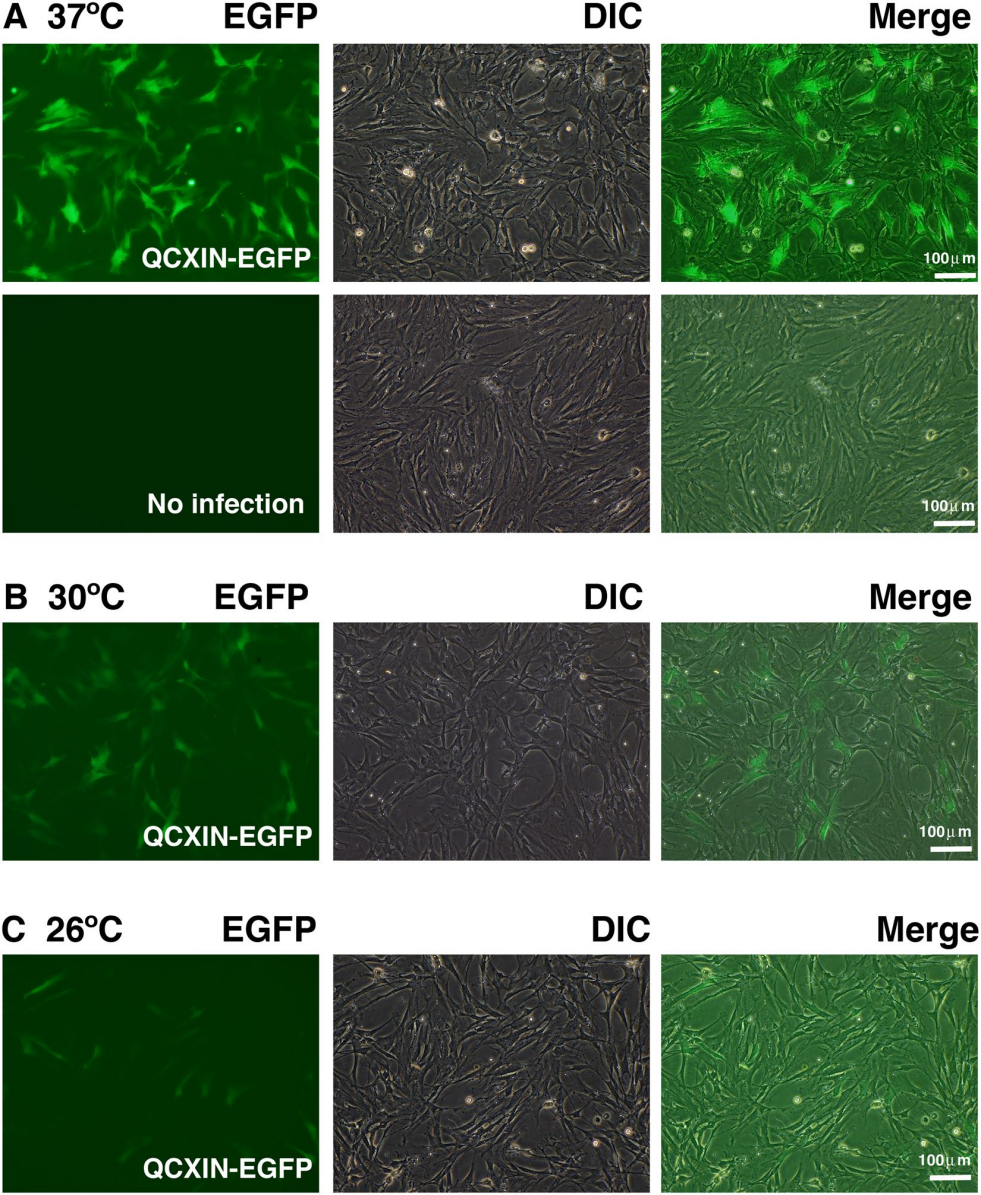
Introduction of enhanced green fluorescence protein (EGFP)-expressing retrovirus into primary cells of the loggerhead sea turtle. Left panels display EGFP detection of retrovirus-infected cells. Middle panels display cell morphology of retrovirus-infected cells, viewed under a differential interference contrast (DIC) microscope. Right panels display merged images of DIC and fluorescence images. Results with infection at 37 °C (**A**), 30 °C (**B**) and 26 °C (**C**).

To our knowledge, no previous study has described gene transfer into reptile-derived cells. In the present study, we used enhanced green fluorescence protein (EGFP)-expressing vectors. First, we attempted to introduce the EGFP gene using the lipofection method. However, few cells showed a positive EGFP signal (Figure S1A,B). For the establishment of K4DT method based cells, three genes—mutant CDK4, Cyclin D and TERT—must be transferred into cells (i.e., triple gene transfer). Next, we attempted to introduce the EGFP gene using retrovirus, which carries VSV-G as the envelope glycoprotein. Interestingly, loggerhead primary cell lines that were infected with the EGFP-expressing VSV-G retrovirus showed strong green fluorescence (Fig. 2A). The infection efficiency changed depending on the temperature (Fig. 2A–C) with maximum efficiency of approximately 36% obtained at 37 °C. We concluded that the VSV-G containing retrovirus is the most appropriate vector to establish recombinant loggerhead-derived cell lines.

### Establishment of mutant CDK4-, Cyclin D-, and telomerase-expressing loggerhead-derived cell lines with normal karyotype

**Figure 3:**
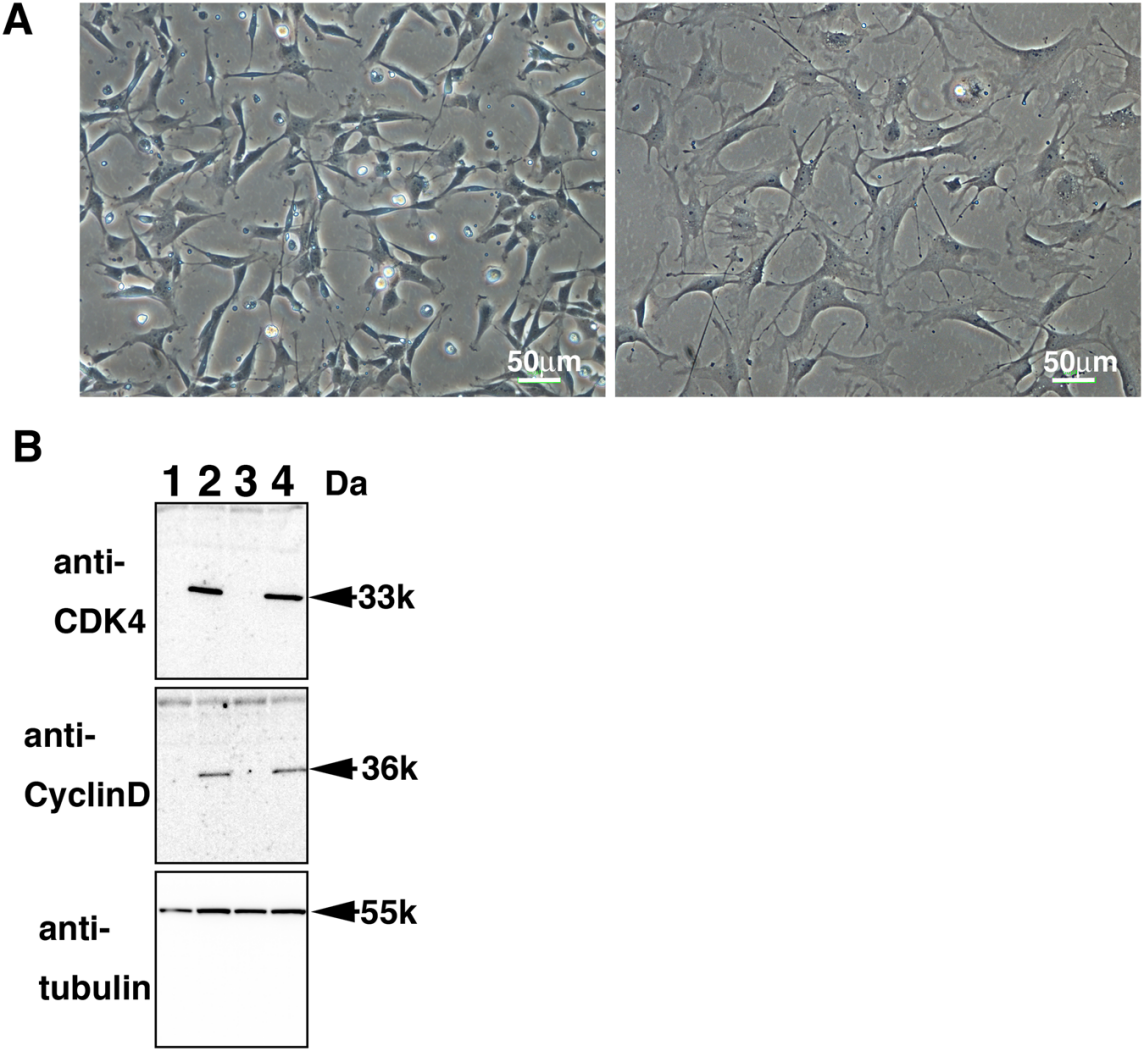
Establishment of Cyclin-dependent kinase 4 (CDK4)-, Cyclin D- and telomerase-expressing cells derived from the loggerhead sea turtle. (**A**) left panel, cell morphology of the loggerhead-derived cells, expressing CDK4, Cyclin D, and telomerase. Right panel, cell morphology of the loggerhead primary cells. (**B**) Protein expression of the introduced CDK4 and Cyclin D. The expression of human CDK4, human Cyclin D, and endogenous tubulin were detected by using western blot analysis. Lane 1, primary A634 cell line; Lane 2, A634-K4DT cell line; Lane 3, primary A640 cell line; Lane 4, A640-K4DT cell line.

We introduced the human-derived mutant CDK4, Cyclin D and TERT into primary cell lines from the loggerhead sea turtle using the recombinant VSV-G retrovirus. We observed that size of infected loggerhead-derived cells were smaller than that of primary cell lines (Fig. 3A), but they had a similar morphology. The smaller size of the recombinant cells likely reflected the more rapid rate of cell proliferation. We detected protein expression of the introduced genes (CDK4 and Cyclin D) using western blot analysis (Fig. 3B). Both recombinant cell types (A634-K4DT and A640-K4DT) displayed high expression levels of CDK4 and Cyclin D, indicating the efficient introduction of their genes into the primary cell lines.

**Figure 4:**
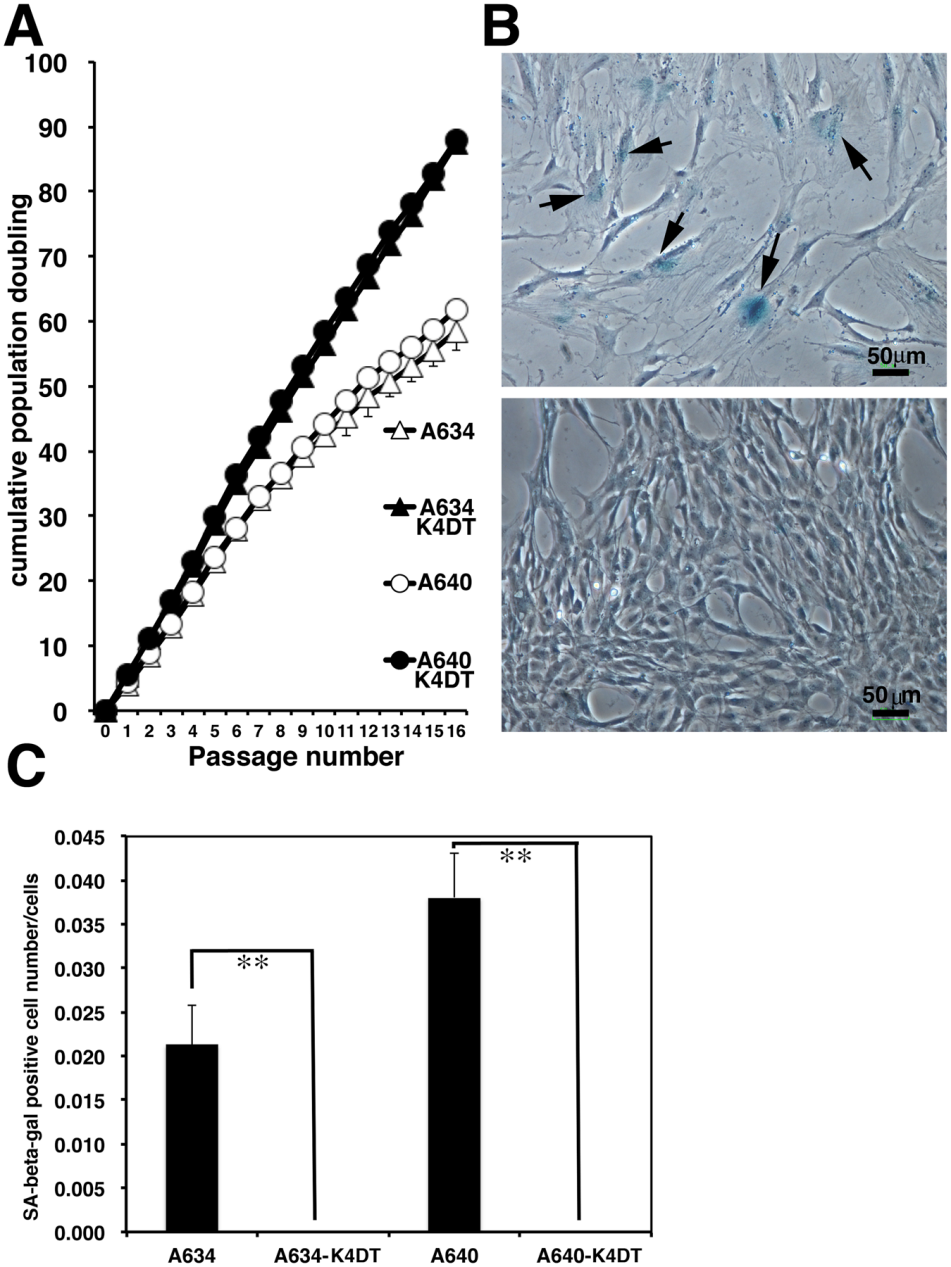
Growth characteristics and cellular senescence of primary and recombinant cells derived from the loggerhead sea turtle. (**A**) Sequential passage and cell growth of primary A634 cells, recombinant A634 derived K4DT cells, primary A640 cells and recombinant A640 derived K4DT cells. At the start of each passage, 5 × 10^4^ cells of each cell line were added to triplicate cell culture wells. (**B**) Detection of senescence-associated β-galactosidase staining in primary A634 cells (upper panel) and A634 derived K4DT cells (lower panel) after 16 passages. The arrows indicate positive blue staining, denoting cellular senescence. (**C**) Incidence of positively stained primary A634 cells, recombinant A634 derived K4DT cells, primary A640 cells and recombinant A640 derived K4DT cells. The percentage of positively stained cells counted in six randomly selected microscopic fields is shown in the graph. The double asterisks indicate statistical significance at the 1% level.

Next, we evaluated the effect of mutant CDK4, Cyclin D and TERT in the loggerhead-derived cell lines. Cell proliferation was considerably enhanced by the introduction of these genes (Fig. 4A). On average, 5 × 10^4^ A634 derived K4DT or A640 derived K4DT cells achieved confluent growth by approximately 2 weeks after seeding in 35 mm cell culture dishes. We compared the cell proliferation speed within 16 sequential passages, at approximately 32 weeks (8 months) of the experimental period. The recombinant cells did not exhibit a slowdown of cell proliferation, whereas the primary cell lines showed delayed proliferation by the end of the experiment (Fig. 4A). The primary cell lines displayed an enlarged cell cytoplasm and the cells stained positively with senescence-associated beta-galactosidase (SA-β-gal) after 16 passages, indicating that these cells had senesced (Fig. 4B). On the other hand, the recombinant cells showed no morphological changes after long-term passage and did not stain positively with SA-β-gal (Fig. 4C).

To test the chromosome number and banding pattern of the established recombinant loggerhead-derived cell lines, we used two cell lines from different individuals to evaluated the condition of each chromosome (Figure S2A-C). Several mitotic cells displayed variation in chromosome number (Figure S2C, A634-K4DT). However, this type of chromosome variation was also observed in wild-type fibroblast cell lines (Figure S2C,634). Therefore, we concluded that more than 90% of the recombinant cells (A634-K4DT and A640-K4DT) derived from loggerhead sea turtle maintained the intact chromosome number and diploid nature.

**Figure 5:**
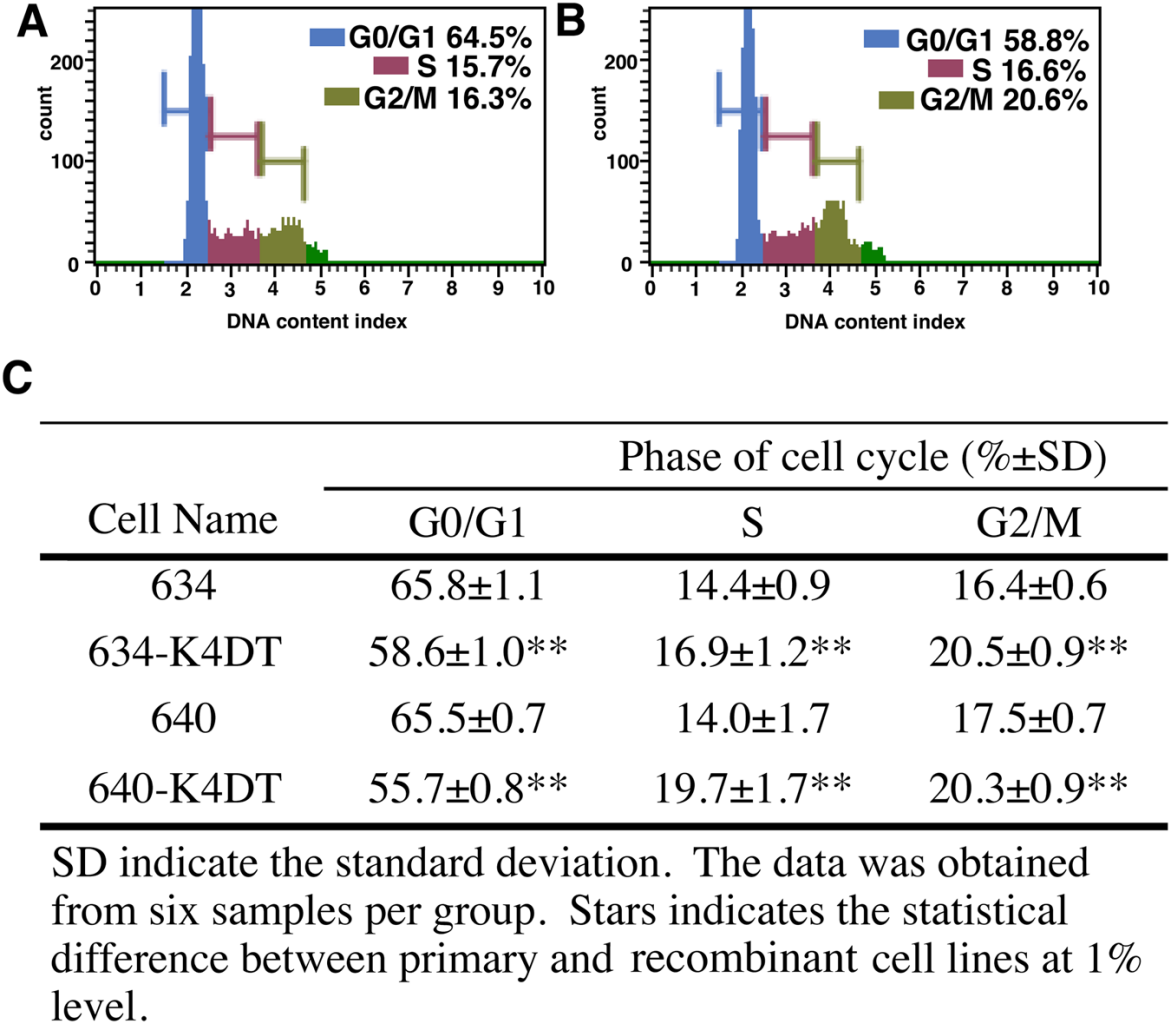
Cell cycle analysis of primary and recombinant cells derived from the loggerhead sea turtle. A and B, Histograms obtained from the cell cycle analysis: (**A**) primary cells (A634); and (**B**) recombinant cells (A634 derived K4DT). The percentages of cells in the G0/G1, S and G2/M phases are shown in color. (**C**) Percentages of primary and recombinant loggerhead-derived cells at each cell cycle phase. The data were obtained from five replicate samples. The double asterisks indicate statistical significance at the 1% level.

Analysis of the cell cycle of the original and established fibroblast cell lines (Fig. 5A,B) revealed that loggerhead-derived cell lines proliferated more slowly than mammalian-derived cells (compared with the proliferation of mouse embryonic fibroblasts)^20^. In agreement with this prediction, most of the cells remained in the G0/G1 phase (Fig. 5A). The established recombinant loggerhead-derived cells showed a significantly lower percentage of cells in the G0/G1 phase (55.7% versus 65.5%), and a higher percentage of cells in the S phase (19.7% versus 14.0%) and G2/M phase (20.3% versus 17.5%) (Fig. 5C). The results indicated that the progression speed of the cell cycle of established recombinant cell line was accelerated compared to that of wild-type cells.

**Figure 6:**
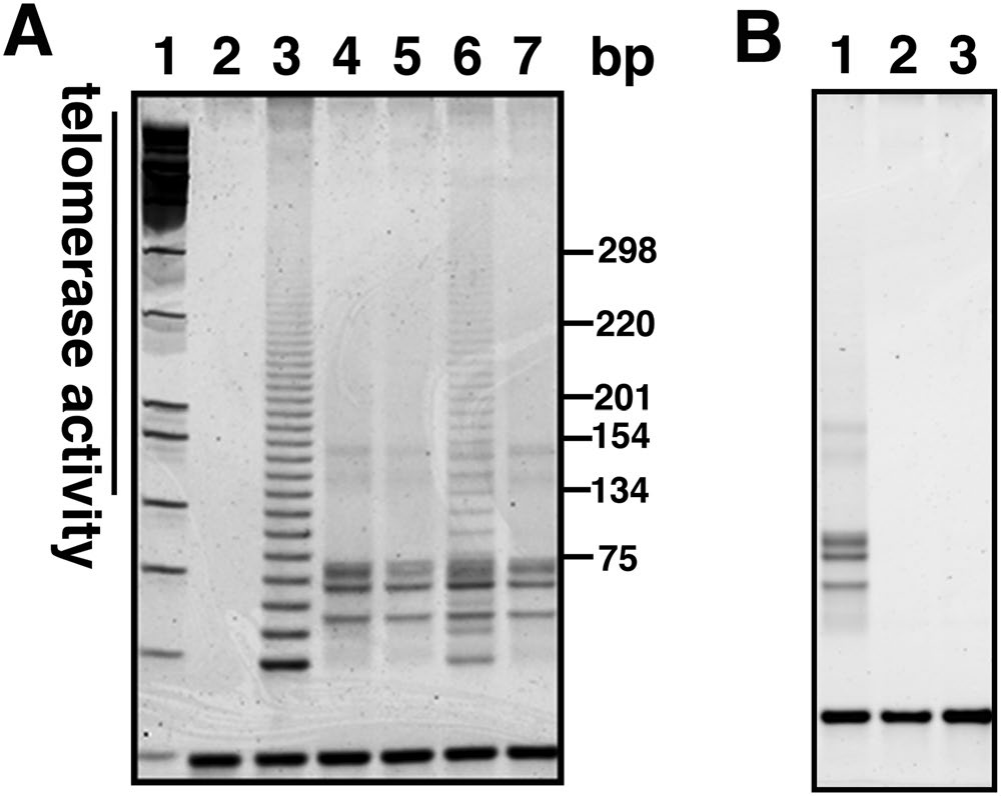
Detection of telomerase activity using stretch PCR. (**A**) Results of primary and recombinant sea turtle derived cell, Lane 1, Molecular weight marker, Lane 2, negative control, Lane 3, positive control sample (HeLa cells), Lane 4, A634 primary cells, Lane 5, A634 derived K4DT cells, Lane 6, A634 derived K4DT + TERC, Lane 7, A634 derived K4D cells. (**B**) Detection of telomerase activity with protein lysate (Lane 1), heat inactivated A634 derived protein (Lane 2), RNase treated A634 derived protein (Lane 3).

As the next stage, we evaluated the activity of telomerase, an enzyme that extends the repeat sequences at the ends of chromosomes. Telomerase activity is reportedly essential for cellular immortalisation. For the accurate evaluation of telomerase activity, we used the stretch PCR assay, which is improved, and more specifically detects telomerase activity^21^. The established loggerhead-derived cell lines did not display telomerase activity sufficient to show the ladder DNA pattern in the assay (Fig. 6A, lane 4). However, it cannot be excluded that the pattern was present but masked by the dirty background. A previous study determined that the expression of two components of telomerase—telomerase RNA component (TERC) and telomerase Reverse transcriptase (TERT)—are required for telomerase activity in three equid species^22^. We suspected that human TERT might not form the functional enzymatic complex with endogenous loggerhead derived TETC, which could potentially result in a lack of telomerase activity in our established A634-K4DT cell line. To evaluate this possibility, we additionally introduced human TERC into loggerhead-derived cells expressing mutant CDK4, Cyclin D and TERT (A634K4DT + TERC). We prepared the recombinant lentivirus for human TERC expression. The lentivirus contains an expression cassette of the resistant gene against G418. As expected, loggerhead cell lines infected with the TERC expressing lentivirus showed resistance to G418 selection (Figure S3), suggesting the successful transfer of the TERC expression cassette. Furthermore, the stretch PCR assay showed that recombinant loggerhead cell lines expressing mutant CDK4, Cyclin D, TERT and TERC are positive for telomerase activity (Fig. 6, lane 6). Interestingly, we observed the enzymatic activity to extend the telomere repeat sequence, the intensity of DNA ladder was much weaker than that of the positive control (HeLa cell; Fig. 6, lane 3). We concluded that the loggerhead-derived fibroblast cell line transfected with both telomerase-associated genes, TERT and TERC, has enzymatic activity of telomerase, although its activity is relatively low. To address the dirty background of the primary loggerhead sea turtle cell extract observed in Lane 4 of Fig. 6A, protein lysates were heat inactivated or treated with RNase. As shown in Fig. 6B, the cell extract from the primary cell line displayed reproducible dirty background signals (Fig. 6B, lane 1). The background signals completely disappeared after the heat denaturation (Fig. 6B, lane 2) or RNase treatment (Fig. 6B, lane 3).

**Figure 7:**
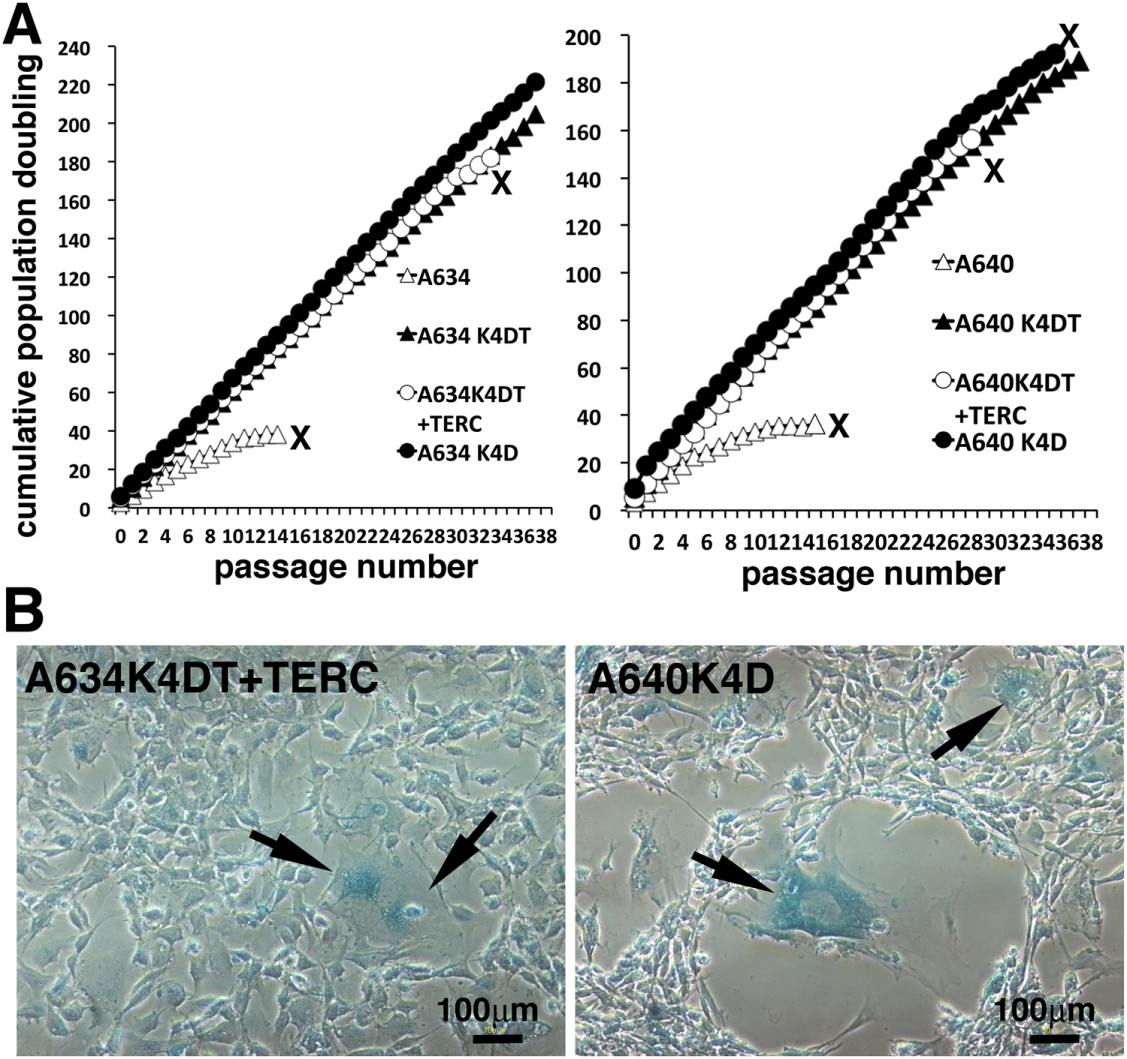
Sequential passage experiments with primary and recombinant cells. (**A**) Left panel, Results of sequential passages of A634 derived primary, A634 derived K4DT, A634 derived K4DT + TERC and A634 derived K4D cells. Right panel, Results of sequential passages of A640 derived primary, A640 derived K4DT, A640 derived K4DT + TERC and A640 derived K4D cells. X indicates the termination of cell growth. (**B**) Left panel, senescence-associated β-galactosidase staining of A634 derived K4DT + TERC cells at passage 36. Right panel, senescence-associated β-galactosidase staining of A640 derived K4D cells at passage 38. Arrows indicates the positively stained cells.

Finally, we evaluated the effect of the expression of mutant CDK4, Cyclin D, TERT and TERC with sequential passage again. In the second round of the sequential experiments, we focused on the cell proliferation of the recombinant cell line that expressed mutant CDK4 and Cyclin D (K4D cells). As shown in Fig. 7A, we evaluated proliferation of cell lines derived from two loggerhead sea turtles (A634 and A640). Furthermore, we evaluated the cell proliferation of primary fibroblast cell line, K4DT cell line, K4DT + TERC cell line and K4D cell line. As shown in Fig. 7A, primary fibroblast cell line from A634 and A640 stopped cell proliferation at an approximate population doubling (PD) value of 40, which is consistent with the results shown in Fig. 4. Although the recombinant cell lines showed continuous cell proliferation, the A634 derived K4DT + TERC cell line unexpectedly stopped proliferating at passage 33, and A640 derived K4DT + TERC cell line also unexpectedly stopped proliferating at passage 28. Furthermore, the A640 derived K4D cell line also stopped cell proliferation at passage 35, indicating that expression of mutant CDK4 and Cyclin D does not support the immortal growth of the loggerhead sea turtle cell lines, although growth for over 30 passages can be practically considered close to immortalisation. We maintained the rest of cells up to passage 38. Then, we unfortunately had outbreak contamination yeast into cell culture dishes, and finally ended the sequential passage experiment at 2 years, 6 months. To search for evidence of cellular senescence, we stained A634 derived K4DT + TERC cell line at passage 34 (Fig. 7B, left panel) for SA-β-galactosidase and stained A640-derived K4D cell line at passage 38 (Fig. 7B, right panel). Both cell lines were positive for cellular senescence. We concluded that although the expression of mutant CDK4 and Cyclin D induces dramatically extended cell proliferation until senescence, which might practically be close to immortalisation, the cell lines are not fully immortalised in the exact terminology of cell biology.

## Discussion

In the present study, we optimized the culture conditions to establish primary cell lines from the loggerhead sea turtle using DMEM/F12 medium. We obtained primary cell lines using other cell culture media, including DMEM and F12. We previously demonstrated that RPMI 1640 provided optimal conditions for primary cell lines of the hawksbill turtle^18^. Thus, the optimal conditions for obtaining primary cell lines may vary according to the species of sea turtle.

We further showed that, under the optimal conditions, primary cell lines could be obtained from cryopreserved tissues, even after 6 months of cryopreservation. In the field of wildlife science, it is sometimes difficult to initiate laboratory investigations immediately after sampling. In fact, we experienced a lack of success when the primary cell culture from the muscle tissues of the Amani rabbit (*Pentalagus furnessi*), a critically endangered animals in Japan. The sampling site was located more than 1,000 km from the laboratory. We could not obtain the successful results of primary cell culture, due to the autolysis of the tissue during transport to the laboratory, even though we kept the tissue in the complete medium (data not shown). Based on this experience, the stable condition under the cryopreservation might be effective for the primary cell culture from critically endangered species.

We demonstrated that the chromosome number of the loggerhead sea turtle is 2*n* = 56. This chromosome pattern was consistent for multiple individuals. Our results differ from those of Nakamura, who reported on the chromosome pattern of the loggerhead sea turtle tissues and embryos in 1949^17^. Nakamura reported that chromosome number of loggerhead was 2*n* = 58^17^. On the other hand, the chromosome number of the green sea turtle (*Chelonia mydas*)^23^, olive ridley sea turtle (*Lepidochelys olivacea*)^24,25^ and the hawksbill sea turtle (*Eretmochelys imbricata*)^16^ were consistently reported to be 2*n* = 56. Hybridisation between multiple strains of sea turtle, including the loggerhead, has previously been demonstrated^4,26–29^. If the chromosome number of the loggerhead differs from that of other sea turtle strains, then the offspring of hybridisation cannot stably survive in the field. Variations in chromosome structure and chromosome number restrict chromosome stability, and infertile phenotypes have occasionally been observed in the inter-crossing between two species with different chromosome numbers, for example, the donkey and horse^30^. In the present study, we demonstrated that the loggerhead sea turtle has a 2*n* = 56 chromosome number, which is identical chromosome number to other sea turtle strains. The results imply that an identical chromosome number is the primary reason for intensive and stable hybridisation in the field. Sea turtle strains have been established and maintained as independent species more than millions of years. However, the intensive hybridisation among multiple species in recent decades indicates that the pure strains of sea turtle are at imminent risk of extinction. The present results imply that an identical chromosome pattern is the primary reason for intensive and stable hybridisation in the field. Further studies are essential to clarify the mechanism how independent species of sea turtle were maintained during evolution, and to develop improved strategies for conservation of pure strains of the sea turtle. As the potential reason, we can listed the quite biased female and male ratio in the section of the introduction^6^. There is a possibility that biased sexual ratio might decrease the mating opportunity among the pure sea turtle strain, resulting in the enhanced hybridization.

We established recombinant sea turtle cell lines containing introduced human homologous genes. To our knowledge, our study is the first to show transgenesis of exogenous genes into reptile-derived cells. The efficiency of gene delivery using the retrovirus was markedly lower when the virus infection was conducted at 26 °C, which is the optimal temperature for primary cell culture of the sea turtle. On the other hand, at 37 °C for 12–20 h, the efficiency of gene delivery markedly increased. The higher temperature was probably required for the efficient enzymatic activity of integrases of the retrovirus and lentivirus.

**Figure 8:**
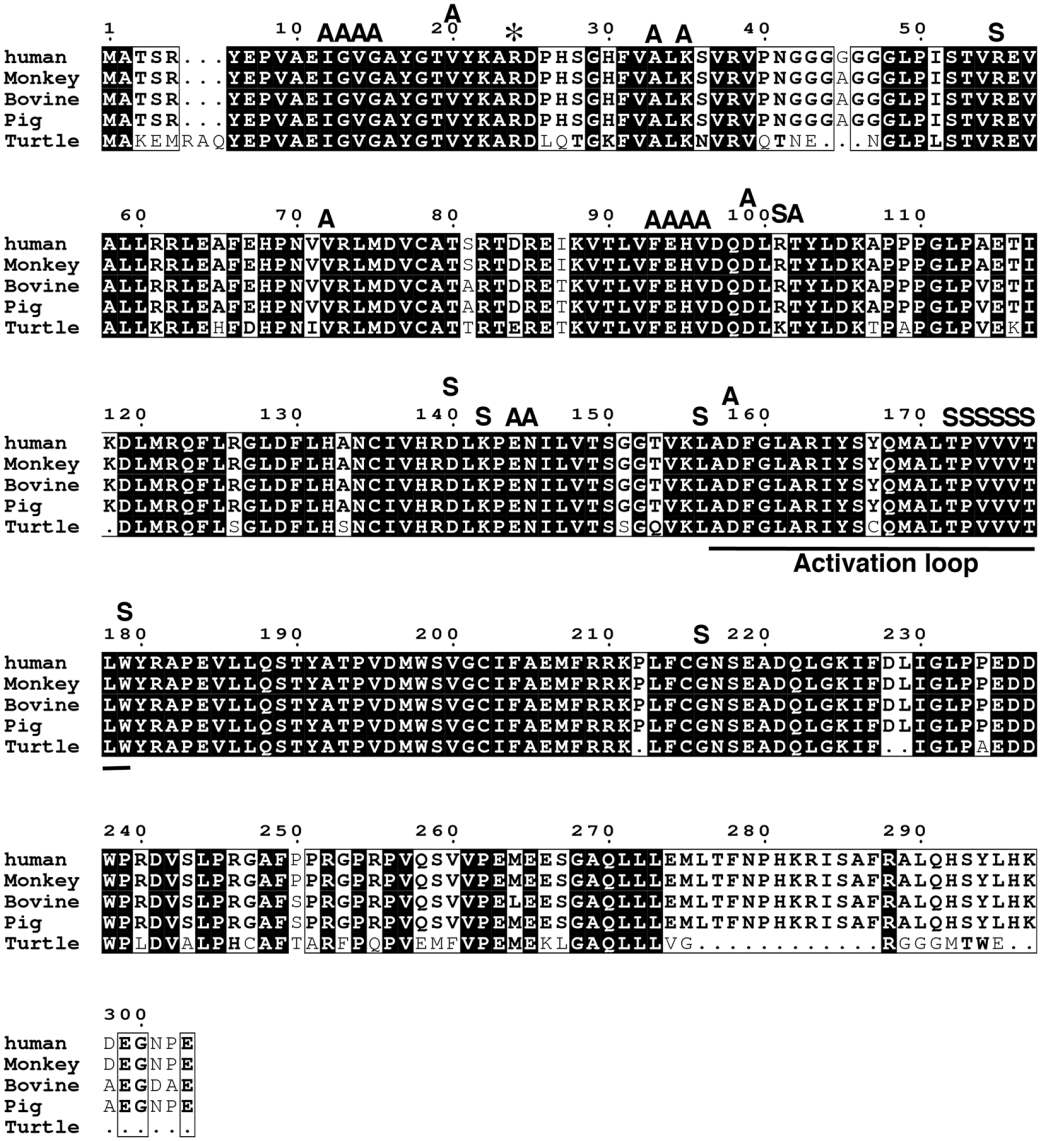
Multiple alignments of CDK4 protein among humans, monkeys, bovines, pigs, and turtles. The amino acid sequences were obtained from the UCSC Genome Bioinformatics database (http://www.ucsc.edu). The conserved amino acids are highlighted with black boxes. The functional motifs corresponding to the ATP-binding domain (**A**) and substrate-binding domain (S) are marked on the sequences. The core amino acid for the binding to p16 protein (24 R) is highlighted with an asterisk.

**Figure 9:**
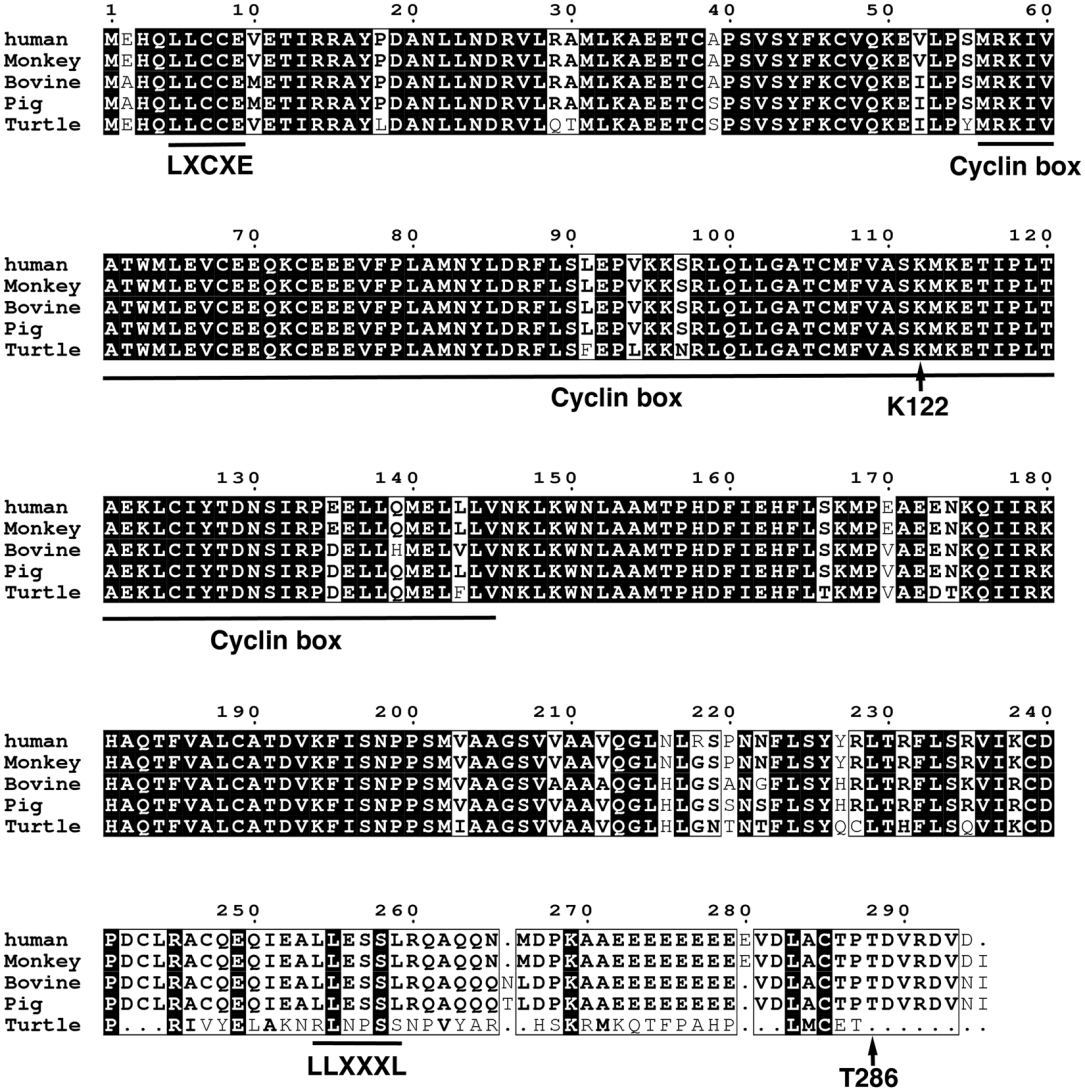
Multiple alignments of Cyclin D protein among humans, monkeys, bovines, pigs and turtles. The amino acid sequences were obtained from the UCSC Genome Bioinformatics database (http://www.ucsc.edu). The conserved amino acids are highlighted with black boxes. The functional motif of LXCXE (which is important for the binding with pRB), the Cyclin kinase box, and the LLXXXL motif (which is important for the binding with steroid receptor co-activators) are underlined.

**Figure 10:**
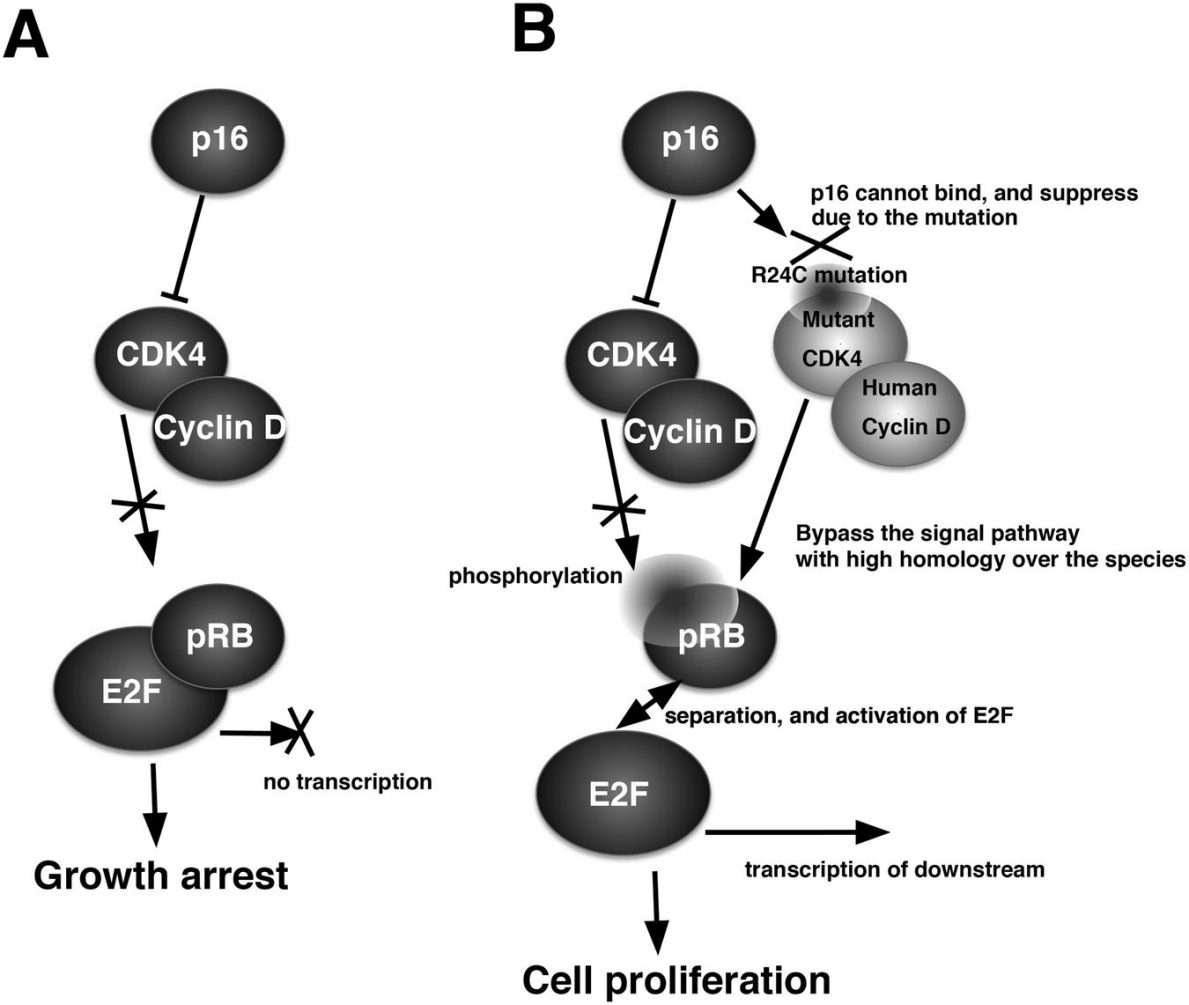
Expected accelerated cell growth mechanism of mutant human-derived CDK4, Cyclin D, and TERT over the multiple species. (**A**) Cell growth arrest under the cellular stress. The protein level of p16 increases under the senescence. The p16 protein will bind to the pocket of the CDK4, and negatively regulates the activity of CDK4-Cyclin D complex. The inactivated CDK4-Cyclin D complex cannot induce the phosphorylation of pRB and its inactivation. Under the intact condition of pRB, E2F is not released from the binding status, resulting in no transcription of the downstream and growth arrest of the cells. (**B**) Enhanced cellular proliferation with mutant CDK4, Cyclin D and TERT. Due to the R24C mutation of mutant human-derived CDK4, p16 protein cannot suppress the activity of protein complex of mutant CDK4 and human Cyclin D. The exogenously introduced human-derived CDK4-Cyclin D complex has high homology with the endogenous CDK4-Cyclin D, and can form the protein complex with endogenous pRB, and phosphorylation. Due to the phosphorylation and inactivation of pRB, the transcription factor, E2F, would be released from the complex and induce cell proliferation.

It has been reported that transduction of human-derived mutant CDK4, Cyclin D and TERT enhances the rate of cell proliferation. Sasaki *et al*. demonstrated the immortalisation of human ovarian surface epithelial cells by using a combination of CDK4, Cyclin D and TERT^31^. Shiomi *et al*. showed that immortalised human myogenic cells maintained their original nature as myogenic stem cells, with the intact chromosome condition^10^. We previously demonstrated the immortalisation of fibroblast cell lines derived from bovine, porcine^9^ and non-human primate species^11^, midget buffalo^14^, prairie vole^12,13,32^, bovine colon epithelial cell^8^ by transgene expression of human mutant CDK4, Cyclin D and TERT. The success of the immortalisation relies on the conserved homology of the cell cycle regulators, CDK4 and Cyclin D, over multiple species. In this study, we compared the homology of the amino acid sequences among multiple species, including humans, monkeys, bovines, pigs and turtles (Figs 8 and 9). The cDNA sequences of CDK4 and Cyclin D are not listed in the genome database for painted turtle. However, we determined the predicted homologous genomic sequences of turtle CDK4 and Cyclin D, based on a homology search with the amino acid sequence of zebrafish. In CDK4, the core position (arginine at 24, asterisk in Fig. 8) for the binding with p16 protein was completely conserved in all investigated species, including turtles. Furthermore, other functional-related amino acid residues (ATP binding motif, A in Fig. 8, and Substrate binding motif, S in Fig. 8) of CDK4 were highly conserved among all of the investigated species. The Cyclin kinase box (amino acids 55–145) showed high homology among the multiple species. Overall, there was an 81% homology of CDK4 between humans and turtles (Fig. 8). In case of Cyclin D (Fig. 9), the Cyclin kinase box (amino acids 550-145) displayed high homology among the multiple species. The mechanism to accelerate the turn over of the cell cycle is summarised in Fig. 10. In the primary cells, the amount of negative cell cycle regulator, p16, is increased because of various cell stresses. The p16 protein binds to CDK4, and negatively regulates the CDK4–Cyclin D complex. The suppression of the CDK4–Cyclin D complex by p16 inhibits the induction of phosphorylation and inactivation of pRB, thereby preventing the transcription of E2F transcriptional factor, and resulting in growth arrest of the host cell (Fig. 10A). However, in the presence of mutant human-derived CDK4 and Cyclin D, endogenous p16 cannot bind to mutant CDK4 because of the point mutation at the binding site with p16 (R24C). Therefore, mutant CDK4 is free from the negative regulation of p16. Thus, the mutant CDK4–Cyclin D complex induces phosphorylation of RB protein in the host cell, based on the high homology among multiple species. In accordance with this notion, we observed that in Cyclin D, the LXCXE motif—which is important for the binding with RB protein—was conserved in all species (Fig. 9)^33,34^. Conserved LXCXE in Cyclin D suggests that the introduced human Cyclin D inactivates the loggerhead-derived endogenous pRB via this motif. The inactivation of pRB results in the separation of transcriptional factor, E2F, and induces cell proliferation. The present results indicate that the cell cycle regulation of the G1/S phase, and especially regulation of the pRB protein-related mechanism, is evolutionally conserved between mammals and reptiles. From the viewpoint of animal evolution, the sea turtle has unique characteristics in possessing a shell structure, known as the carapace, which indicates a genetic distance from mammalians to sea turtle. Surprisingly, the negative regulation system of the cell cycle via pRB is evolutionally conserved, even in the unique vertebrate. To our knowledge, our study is the first to demonstrate that human-derived cell cycle-related molecules are functional in reptiles.

The stretch PCR assay showed that the background signal in primary cell protein lysate completely disappeared when the lysate was treated with heat or RNase (Fig. 6B). TERT forms a protein complex with the RNA component of telomerase, TERC. The existence of background signals, which depend on the protein stability and RNA, might suggest the existence of telomere binding protein in the protein lysate of primary loggerhead sea turtle cells.

In the second round of sequential passage experiments, K4DT + TERC cells stopped proliferating first among the recombinant cells, and was common with two independent cell lines derived from A634 and A640 (Fig. 7). These results were quite unexpected for us because K4DT + TERC cells showed positive telomerase activity (Fig. 6). Although K4DT + TERC cells showed enzymatic reactivity in the assay, the intensity of the DNA ladder was faint. The cell culture temperature of sea turtle cells was approximately 26 °C. Due to this lower culture temperature, there is a possibility that the introduced human TERC and TERT might not have worked well as the telomerase enzymatic complex. K4DT + TERC double transgenic cells were selected by exposure to G418 antibiotic selection to ensure the introduction of TERC. In the selection process, the K4DT + TERC cells required additional passages, compared with the K4D cells. These additional cell passages might be the reason why K4DT + TERC cells stopped proliferating among the recombinant cells. It needs to be noted that the loggerhead sea turtle cells were maintained for more than PD value 200, only with the expression of mutant CDK4 and Cyclin D (K4D cell). The length of telomere repeat was reported to be approximately 50 kb^35^, which is 10 times longer than the human repeat. The length of the telomere repeat sequence might help the proliferation of loggerhead sea turtle cells up to PD 200 only with the expression of mutant CDK4 and Cyclin D.

The efficient establishment of cell lines from critically endangered species represents a valuable tool for enhancing progress in biodiversity research. The established cell lines can be shared as research materials among scientists worldwide. Furthermore, the establishment of cell lines will facilitate the investigation of infectious diseases. Infection with the fibropapilloma virus is a serious threat to the survival of sea turtles^2,36^. Furthermore, sea turtle sex is determined by incubation temperature during embryonic development (temperature-dependent sex determination, TSD)^37^. Although the detailed mechanism of TSD is not fully understood, the enzymatic activity of Cytochrome P450 aromatase, also known as cyp19a1 or aromatase, is temperature sensitive. Aromatase irreversibly catalyses androgens into estrogens, have a critical role to determine the sex of the turtle^38^. Our established cells might contribute to determine the detailed molecular information how aromatase activity sea turtle would be affected by the temperature, and the understanding of TSD mechanism. The application of cell cycle-related molecular genetics to wildlife science will facilitate the elucidation of the underlying history of animal evolution.

## Materials and Methods

### Tissue sampling

The loggerhead sea turtles were maintained as a closed colony at the Port of Nagoya Public Aquarium, Minato-ku, Nagoya, Japan. During the labelling of the sea turtles with identification tags (at approximately 1–2 years of age; see Fig. 1 of the reference), we obtained small (3 mm × 3 mm) dermal-tissue biopsies from the flipper-like fins^39^. All biopsy processes were authorized by a veterinary doctor affiliated with the Port of Nagoya Public Aquarium. The experimental protocol and procedure were approved by the committee of animal handling at the Port of Nagoya Public Aquarium. The biopsy samples were immediately immersed in DMEM/F12 medium (Life Technologies, Carlsbad, CA, USA; product code, 10565-042) containing 10% FCS, and 1× antibiotics (Nacalai Tesque, Kyoto, Japan; product code, 02892-54). For optimization of the cell culture conditions, we used four different media: DMEM (Nacalai Tesque; product code, 845935), DMEM/F12 (Life Technologies), Ham’s F12 (Wako Chemicals, Osaka, Japan; product code, 8708335) and RPMI 1640 (Wako Chemicals; product code, 18902025). Each medium contained 10% fetal calf serum and 1× antibiotics.

### Establishment of primary cell lines from loggerhead sea turtles

Wells of a 6-well cell culture dish were coated with type I collagen (Cellmatrix Type I-A, Nitta Gelatin, Osaka, Japan), diluted 40× with ice-cold PBS, and were treated overnight at 4 °C. For optimization of the primary cell culture conditions, we tested the four medium conditions with the tissues obtained from three sea turtles. We counted the number of cells from the primary tissues on every second day after the start of the cell culture. The cell culture was maintained at 26 °C under 5% CO_2_ in a humidified chamber, with replacement of each medium twice per week. The detailed method for the passage was described previously^39^.

### Tissue cryopreservation

Small pieces of tissue were immersed in 1 mL of cryoprotective medium (Cell Banker, Mitsubishi Chemical Medience Corporation, Tokyo, Japan; product code, 248085) using a cryogenic tube (Thermo Fisher Scientific, Waltham, MA, USA; product code, 124037). The tubes were frozen overnight in a −80 °C freezer with a Mr. Frosty freezing container (Thermo Fisher Scientific; product code, 5100-0001). On the following day, the tubes were transferred to a liquid nitrogen tank for longer preservation.

### Preparation of recombinant retrovirus and infection of primary loggerhead sea turtle cells

Recombinant retrovirus were obtained by transient expression of the appropriate packaging plasmid in 293 T cells. For the expression of human mutant Cyclin-dependent kinase 4 (CDK4)R24C, we used the pQCXIP-CDK4R24C retrovirus plasmid. For the expression of human Cyclin D and TERT, we used the pQCXIN-CyclinD and pCLXSH-ACC-TERT plasmids, respectively. The retrovirus plasmid was introduced into 293 T cells using the lipofection method with two different packaging plasmids: pCL−gp (an expression plasmid with a deletion mutation for the envelope gene of pCL10A1) and pCMV-VSV-G-RSV-Rev (an expression plasmid of the VSV-G envelope protein and Rev). Both plasmids were a kind gift from Dr. Hiroyuki Miyoshi, Riken BioResource Center, Tsukuba, Japan. For optimization of gene transfer, we used QCXIN-EGFP retrovirus. For the efficient immortalisation with three genes, the supernatants of mutant CDK4, Cyclin D and the TERT-expressing recombinant virus were recovered from transfected cells, filtered through 0.45 µm disks (Sartorius, Goettingen, Germany; product code, 17598 K) and mixed. Primary loggerhead sea turtle cells were exposed to the mixed recombinant virus. To increase the infection efficiency, the primary cells were exposed to the recombinant virus overnight at 37 °C. The infected mass population of cells was subjected to further analysis, such as western blotting or growth analysis. No cell cloning or selection was applied to the established cells.

### Western blotting

Protein expression of exogenously introduced CDK4 and Cyclin D was detected using primary antibody against CDK4 (BD Bioscience, Franklin Lakes, NJ, USA; product code, 610147) and primary antibody against Cyclin D (mouse anti-Cyclin D monoclonal antibody; BD Bioscience; product code, 554180), and the corresponding horseradish peroxidase-conjugated secondary antibody. The western blotting procedure was described in detail previously^20^.

### Karyotype analysis

The karyotype analysis of established primary and recombinant cell lines was conducted following a previously described method^25,39^. For the determination of chromosome number and banding pattern, we used two primary cell lines: A634 and A640. We examined 22–24 mitotic cells for wild type. For the determination of the chromosome number and banding pattern of recombinant cells, we analysed 55-56 mitotic cells.

### Population doubling assay

The primary or immortalised cell lines were seeded at a cell density of 5 × 10^4^ cells per 3.5-cm plastic dish. The experiment was performed in triplicate. When one of the cell types reached confluent growth, we treated the primary and recombinant cells with trypsin and counted the total number of cells in each dish using an automated cell counter (Invitrogen, Carlsbad, CA, USA). We evaluated the rate of cell growth by calculating the cumulative population doubling (PD), using the formula PD = log_2_ (A/B), where A is the number of harvested cells at the end of each passage, and B is the number of seeded cells at the starting. We assessed the cell viability by staining the cells with Trypan blue solution.

We repeated the sequential passage experiments twice to ensure a scientific conclusion. In the first round, we tested the four cell lines: A634 primary, A634 K4DT cells, A640 primary and A640 K4DT cells. In the second round, we tested the eight cell lines in total: A634 primary, A634 K4DT, A634 K4DT + TERC, A634 K4D, A640 primary, A640 K4DT, A640 K4DT + TERC, and A640 K4D. We carried out the passage approximately every 14 days up to passage 15, and the passage period became longer at approximately 4 weeks per passage because the cell growth speed slowed in later passages. In the triplicated wells, when one of the well showed less than 5 × 10^4^ cell, which initially seeded at previous passage, we determined that corresponding cell line stopped proliferation.

### Cell cycle analysis

We analysed the cell cycle by fixing 1 × 10^6^ primary or established recombinant loggerhead-derived cells with 4% paraformaldehyde solution in PBS. The fixed cells were stained with the Muse cell cycle assay kit (Merck Millipore, Darmstadt, Germany) and applied to the cell analyser. Data were obtained from five replicate samples and the statistical significance was evaluated using the Mann-Whitney U test.

### Detection of cellular senescence

After 16 passages, the primary cell lines were stained with SA-β-gal, which is a biomarker of cellular senescence. Positive staining was observed by using a senescence detection kit (BioVision, Milpitas, CA, USA). The stained cells were photographed under a TS-100 microscope (Nikon, Tokyo, Japan) equipped with a digital camera. We randomly selected six microscopic fields and counted the number of positively stained cells. Statistical analysis was performed using the Mann-Whitney U test. In the second round of sequential passages, when cells showed enlarged cytoplasm and slow down of cell proliferation, cells were collected, and applied to the detection of the cellular senescence.

### Detection of telomerase activity

The primary and recombinant cells were homogenized in the lysis solution, which was supplied as a component of the telochaser detection kit (TOYOBO, Osaka, Japan), which based on the previously published protocol^21^. Cells (1 × 10^6^) were homogenized in 200 µL of lysis buffer, according to the manufacturer’s protocol. The PCR products were separated by 15% polyacrylamide gel electrophoresis and stained with GelRed (Biotium, Hayward, CA, USA).

### Gene transfer of human telomerase RNA component (TERC) into loggerhead derived cells

For the contraction of expression plasmid for human TERC, we obtained an expression cassette of human TERC (pBS-U3-hTR 500) from Dr. Kathy Colins through Addgene. The cDNA fragment of pBS-U3-hTR (Sal I(blunt)-BamHI) was ligated into the multiple cloning sites of LVSIN-CMV-Neo lentivirus (EcoRI(blunt)-BamHI) (Takarabio). The LVSIN-CMV-TERC Neo plasmid was introduced into 293 T cells for the virus packaging with HIV-gp and pCMV-VSV-G-RSV-Rev plasmids (kindly provided by Dr. Hiroyuki Miyoshi, Riken BioResource Center, Tsukuba, Japan). The detailed method for the lentivirus reparation was previously described by us^40^. A634-K4DT and A640-K4DT cells were infected with TERC expressing lentivirus overnight at 37 °C. After the infection, cells were selected with the selection medium containing 1% FCS and 7 mg/mL G418 (Nacalai Tesque). No cell cloning or selection was applied to the established cells.

### Statistical analysis

For the detection of cellular senescence, and cell cycle analysis, we evaluated statistically significant differences by using the Mann-Whitney U test. We used the computer software KaleidaGraph (Synergy Software, Reading, PA, USA), and a significance level of 0.05%.

## Electronic supplementary material

 Supplementary Information
