# Supplementary material for: Expression of human mutant cyclin dependent kinase 4, Cyclin D and telomerase extends the life span but does not immortalize fibroblasts derived from loggerhead sea turtle (Caretta caretta)
Source: Sci Rep. 2018 Jun 20;8:9229. doi: 10.1038/s41598-018-27271-x (PMC6010431; doi:10.1038/s41598-018-27271-x)
Supplement: Supplementary file 1 — Supplementary Information [file 41598_2018_27271_MOESM1_ESM.pdf]

Expression of human mutant cyclin dependent kinase 4, Cyclin D and telomerase extends the life span but does not immortalize fibroblasts derived from loggerhead sea turtle (*Caretta caretta*)

Tomokazu Fukuda[1,2,3]*, Takahiro Eitsuka[4], Kenichiro Donai[4], Masanori Kurita[5], Tomomi Saito[6], Hitoshi Okamoto[5], Kodzue Kinoshita[7], Masafumi Katayama[3, 8] Hiroshi Nitto[5], Takafumi Uchida[4], Manabu Onuma[3, 8], Hideko Sone[9], Miho Inoue-Murayama[3,7], Tohru Kiyono[10]*

[1]Graduate School of Science and Engineering, Iwate University, Morioka, Japan
[2]Soft-Path Engineering Research Center (SPERC), Iwate University, Morioka, Japan
[3]Wildlife Genome Collaborative Research Group, National Institute for Environmental Studies, Tsukuba, Ibaraki, Japan
[4]Graduate School of Agricultural Science, Tohoku University, Sendai, Miyagi, Japan
[5]Port of Nagoya Public Aquarium, Nagoya, Japan
[6]Usa Marine Biological Institute, Kochi University, Tosa, Kochi, Japan
[7]Wildlife Research Center, Kyoto University, Kyoto, Japan
[8]Ecological Genetics Analysis Section, Center for Environmental Biology and Ecosystem, National Institute for Environmental Studies, Tsukuba, Ibaraki, Japan
[9]Environmental Exposure Research Section, Center for Environmental Risk Research, National Institute for Environmental Studies, Tsukuba, Ibaraki, Japan
[10]Division of Carcinogenesis and Cancer Prevention, National Cancer Center Research Institute, Tsukiji, Chuo-ku, Tokyo 104-0045, Japan

*Correspondence should be addressed to:
Tohru Kiyono MD, Ph.D. and Tomokazu Fukuda, Ph.D.
Tohru Kiyono, MD Ph. D.
Tsukiji 5-1-1, Chuo-ku, Tokyo, 104-0045 Japan
Email: tkiyono@ncc.go.jp
Tomokazu Fukuda, Ph.D.
4-3-5 Ueda, Morioka, Iwate, 020-8551 Japan
E-mail: tomofukuda009@gmail.com

Running head: Conservation of cell cycle regulators from mammalian to reptiles

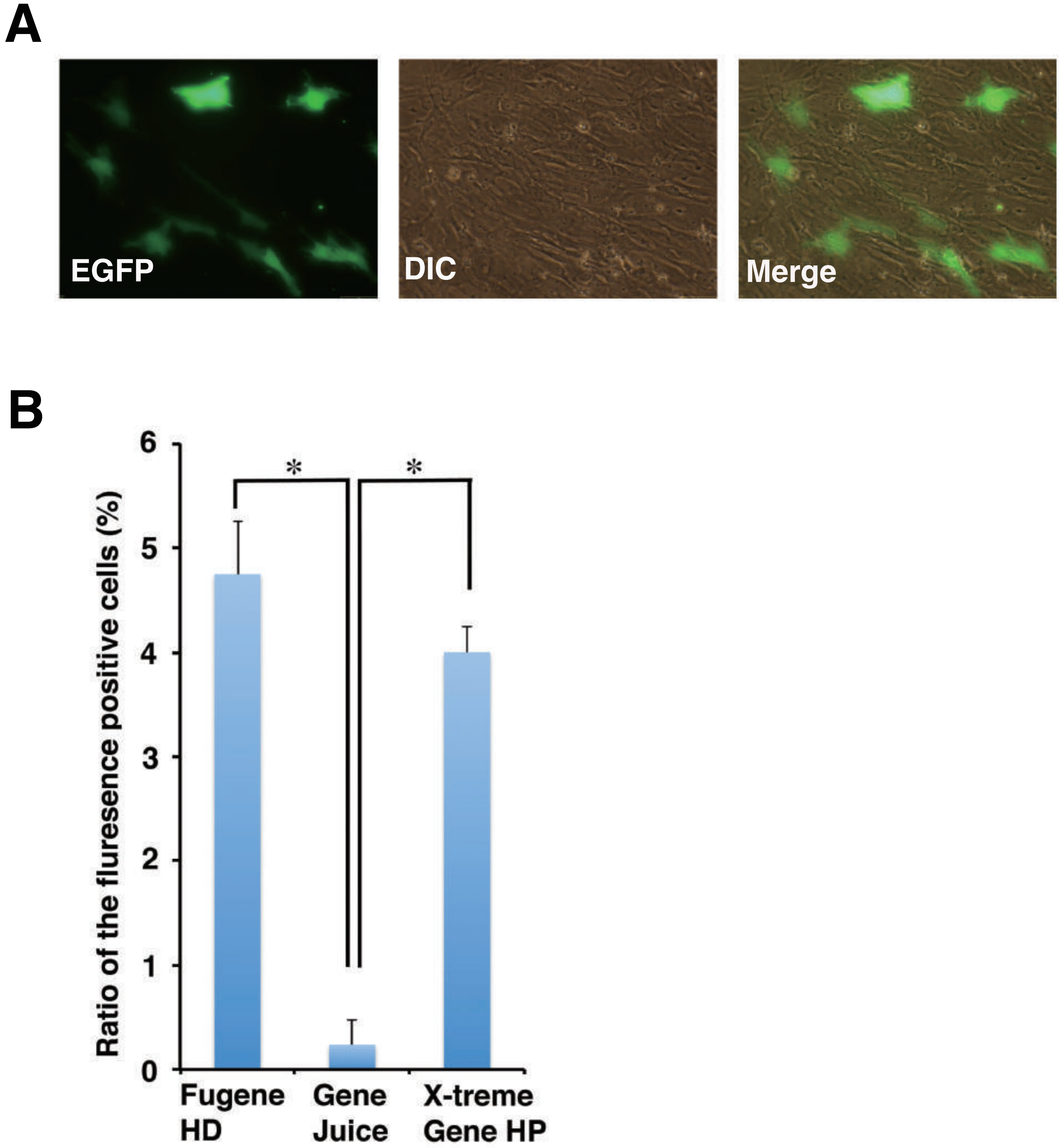
A
EGFP
DIC
Merge
B
Ratio of the fluresence positive cells (%)
0
1
2
3
4
5
6
*
*
Fugene HD
Gene Juice
X-treme Gene HP

A

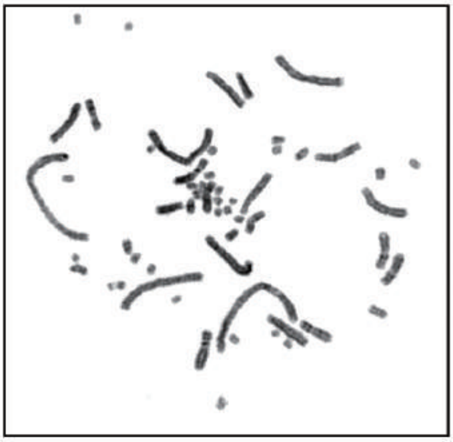

B

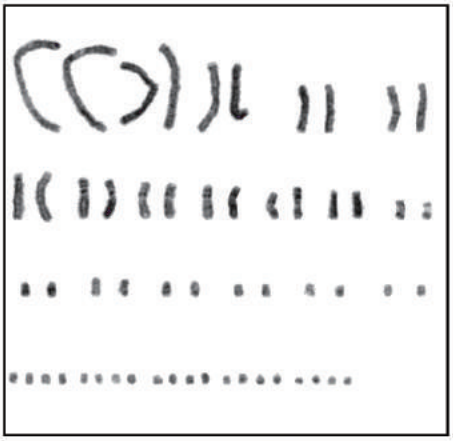

C

Name of Cells ＼ Chrosmosome number	54	55	56	57
634	0	1	20	3
640	0	0	20	2
634-K4DT	1	3	50	2
640-K4DT	0	2	53	0

The summary of the karyotyping analysis of Loggerhead derived primary cells, or immortalized cell lines. The number of mitotic cell, which showed the specific chromosone number were shown in the table. The 22 to 56 mitotic body were analysied.

A

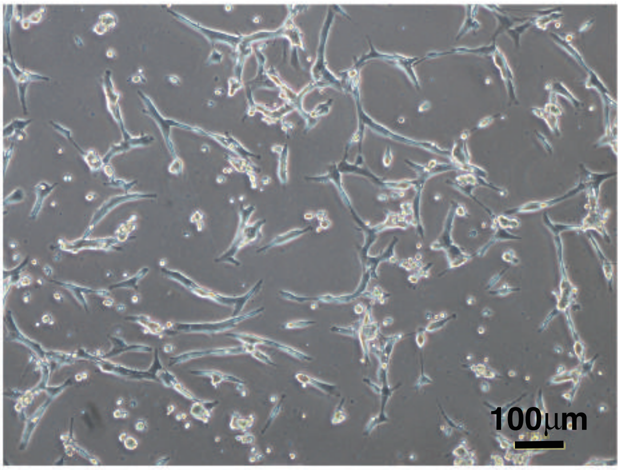

B

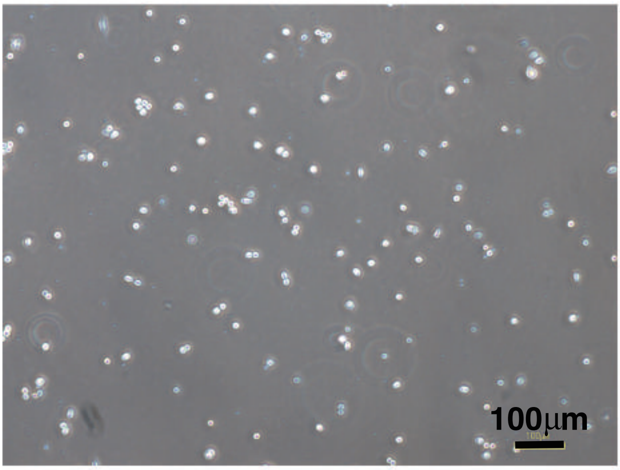

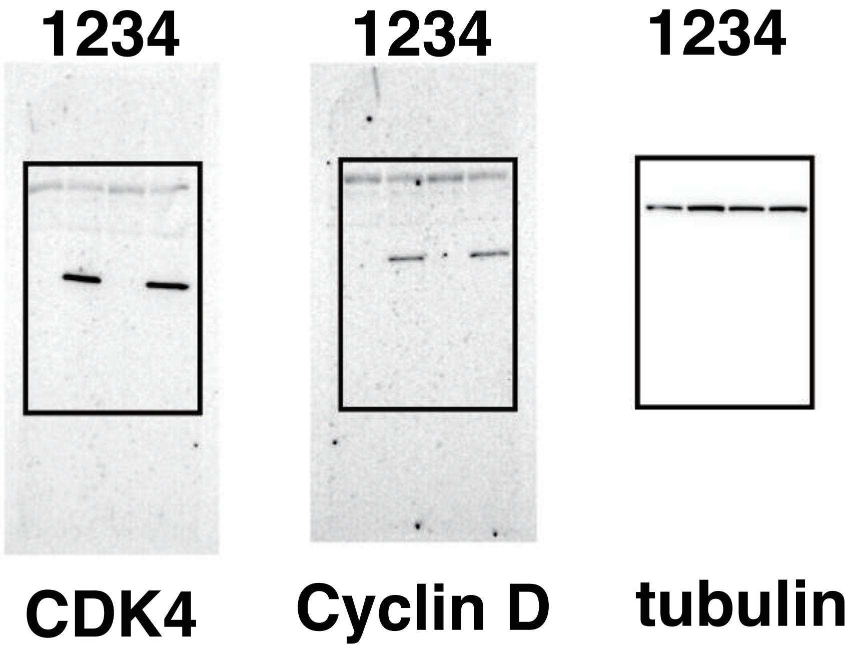

Figure S4: Full length western blots (Figure 3)
Lane1, A634 primary cell, lane2, A634 derived K4DT cell, Lane3, A640 primary cell, Lane4,A640 derived K4DT cell

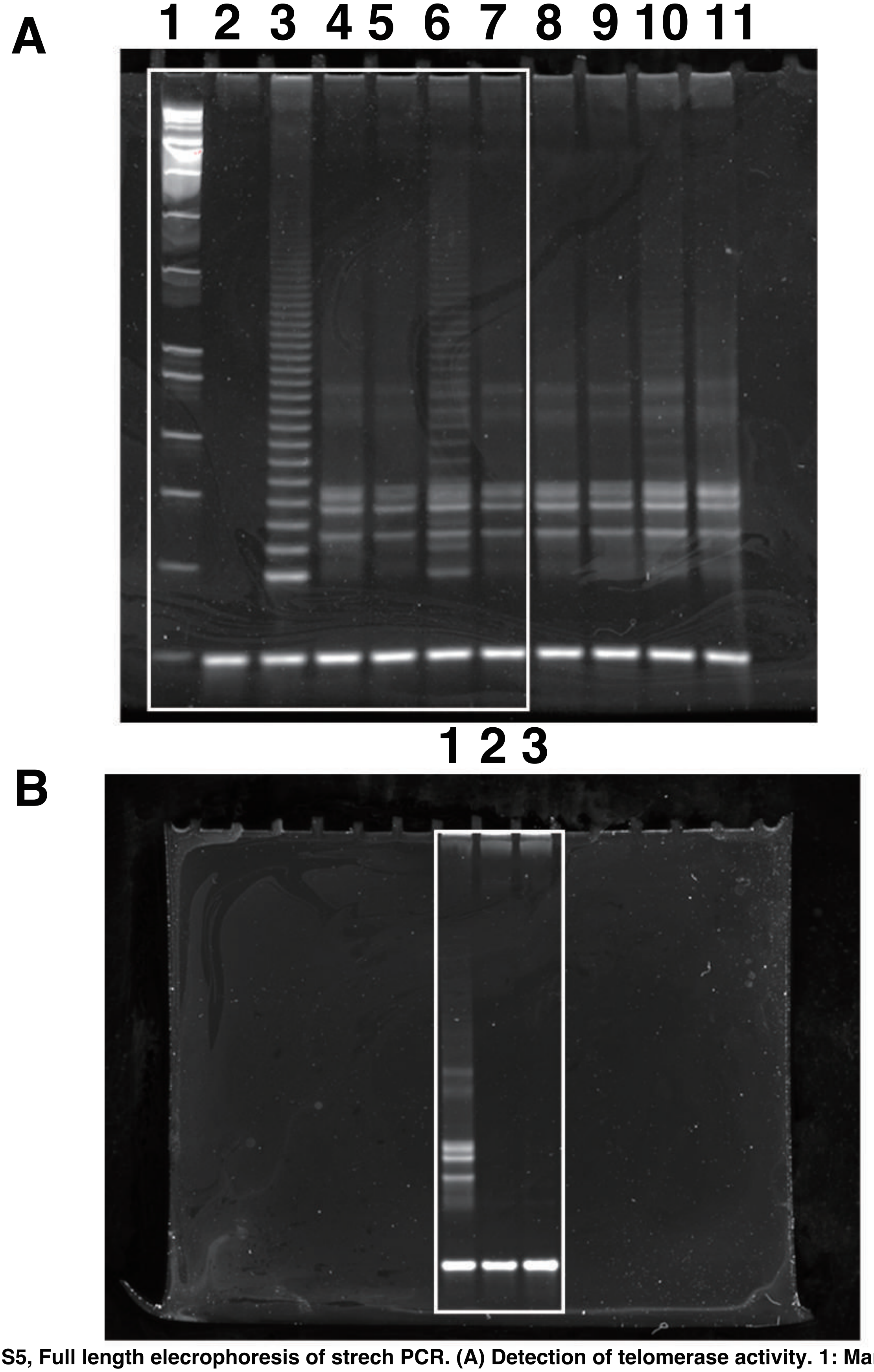

Figure S5, Full length elecrophoresis of strech PCR. (A) Detection of telomerase activity. 1: Marker, 2: negative control, 3: Hela cell(positive control), 4: A634 primary, 5: A634K4DT, 6: A634K4DT+TERC, 7: K4D, 8: A640 primary, 9: A640K4DT, 10: A640K4DT+TERC, 11:A640K4D. (B) Detection of telomerase with primary protein lysate for treated lysate. 1: A634 primary, 2: Heat inactivated A634 primary, 3: RNase trested A634 primry protein

Legends for the Supplemental Figures

Figure S1. Fluorescence detection of EGFP expressing plasmid with the use of various transfection reagents. A, Fluorescence detection of EGFP expressing plasmid in sea turtle cells using Fugene HD. B, Ratio of fluorescence positive cells with three kinds of transfection reagents in sea turtle cells.

Figure S2. Karyotype analysis of A640 derived K4DT cells. A, Mitotic phase of A540 derived K4DT cells. B, Alignment of mitotic chromosomes with G-banding analysis of A640 derived K4DT cells; $2n = 56$. C, Chromosome number of A634 derived primary and K4DT cells, and A640 derived primary and K4DT cells. Twenty to 56 mitotic cells were analyzed.

Figure S3. Antibiotic selection of recombinant cells with G418. A, Cell morphology of recombinant cells expressing TERC and the gene for neomycin resistance. B, Morphology of intact K4DT cells after G418 selection. Note that most of cells

displayed a rounded morphology and were sensitive to G418 selection.

Figure S4. Original full scan blot of Figure 3.

Figure S5. Original full scan of Figure 6.
